# Xq26.3-q27.1 duplication including *SOX3* gene in a Chinese boy with hypopituitarism: case report and two years treatment follow up

**DOI:** 10.1186/s12920-022-01167-2

**Published:** 2022-02-03

**Authors:** Caiqi Du, Feiya Wang, Zhuoguang Li, Mini Zhang, Xiao Yu, Yan Liang, Xiaoping Luo

**Affiliations:** 1 Department of Pediatrics, Tongji Hospital, Tongji Medical College, Huazhong University of Science and Technology, Wuhan, 430030 China; 2 Department of Biology, Boston University, Boston, MA USA; 3 Department of Endocrinology, Shenzhen Children’s Hospital, Shenzhen, 518038 China

**Keywords:** Hypopituitarism, Xq26.3-q27.1 duplication, *SOX3* duplication, Recombinant human growth hormone, Case report

## Abstract

**Background:**

*SOX3* is essential for pituitary development normally at the earliest stages of development. In humans, variants of *SOX3* can cause X-linked hypopituitarism with various clinical manifestations, with or without mental retardation.

**Case presentation:**

We present an 8-year-old Chinese patient with congenital hypopituitarism who had a 6.180 Mb duplication on Xq26.3q27.1 including *SOX3*, *F9*, and eight other contiguous genes. The main complains of the boy was short stature. His height was 90.1 cm (− 5.87SDS), weight 11.5 kg (− 5.25SDS). He developed growth hormone (GH) deficiency, cryptorchidism and low thyroid function. Pituitary magnetic resonance imaging revealed the pituitary dysplasia. After diagnosis, levothyroxine was given for one month first, and the thyroid function basically returned to normal, but the growth situation did not improve at all. Then recombinant human GH was given, his height, growth rate and height SDS were improved significantly in the 2 years follow-up. The level of height SDS improved from − 5.87 SDS before treatment to − 3.27 SDS after the first year of treatment and − 1.78 SDS after the second years of treatment. Gonadal function and long-term prognosis of the patient still need further observation and follow-up.

**Conclusions:**

This is the first case of Chinese male patient with multiple hypophysis dysfunction caused by *SOX3* duplication, which will expand the range of phenotypes observed in patients with duplication of *SOX3*.

## Background

*SOX* (SRY related high mobility group box) genes encode many transcription factors with key regulatory roles in different developmental processes, such as embryogenesis and nervous system development [1]. *SOX3* (OMIM#313430), a member of the *SOX* family, is essential for pituitary development and is expressed in stem and neuroepithelial progenitor cells at the earliest stages of development [2–4].

The variant of *SOX3* often manifested as X-linked hypopituitarism with various clinical manifestations, including isolated GH deficiency (IGHD), congenital hypopituitarism (CH), etc., with or without mental retardation [5]. Studies have identified CH patients in families with GH deficiency and intellectual disability, which further confirms the important role of *SOX3* in the development of the hypothalamic-pituitary axis [6].

Here, we report the first Chinese patient harboring duplication on Xq26.3q27.1 including *SOX3* gene. He developed CH, but he did not present intellectual disability.

## Case presentation

The Chinese boy was eight years old, borned by cesarean section. His birth weight was 2.5 kg, and the length was unknown. After birth, he was diagnosed with hypoxie-ischemic encephalopathy. Therefore, he was hospitalized locally for 40 days. During hospitalization, the fasting blood glucose was normal, the length of penis was not recorded, and no abnormalities were reported in the head magnetic resonance imaging (MRI). Milestones of motor development were normal (sit at 6–7 months, stand alone at 10–11 months and walk at 1.5 years). He had hernia surgery at the age of 4 years. His parents were non-consanguineous and phenotypically normal. Apart from a history of nephrolithiasis, his mother had no other special medical history records. His father is 172 cm tall (− 0.12 SDS), and his mother is 159 cm (− 0.30 SDS) tall. His target height was 171.5 ± 4 cm (− 1.2 SDS). His 1 year-old young sister was normal.

**Fig. 1:**
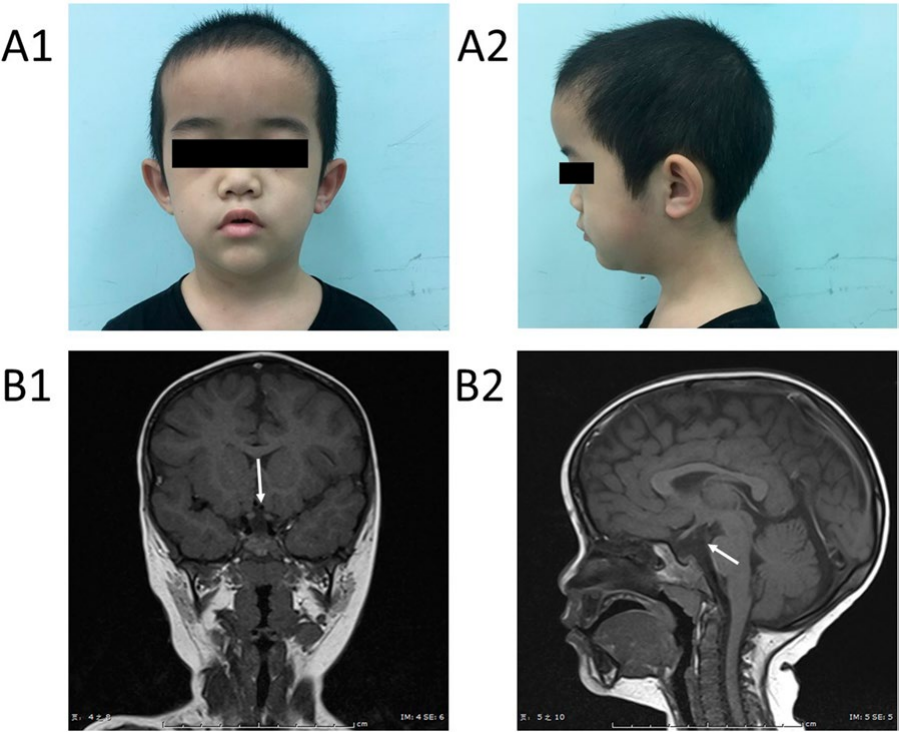
Physical examination results for the 8 -year- old patient. **A1**, **A2** Facial appearance of the patient: slightly larger ears, low ear positions, external rotation, protrusion of ear lobes, thin lips, wide nose bridge, and low forehead hairline. **B1**, **B2** Pituitary MRI of the patient revealed pituitary dysplasia

The main complains of the boy was short stature. His height was 90.1 cm (− 5.87SDS), weight 11.5 kg (− 5.25SDS). He had proportional short stature, slightly prominent forehead, wide eye distance, deep-set eyes, low bridge of nose, hypotrophy of the maxilla, and high and narrow arched palate (Fig. 1A1, A2). He has cryptorchidism on the right side. The left testis was 1 ml and the location was a little bit high. Using the WISC-IV to investigate the intelligence profile, and the results showed that his intelligence was normal (the total IQ was ≤ 92).

Hormone measurement revealed dysfunction of anterior pituitary. The results of GH response on insulin and arginine hydrochloride testing showed peak value of GH as 0.20 ng/mL. His serum concentrations of IGF1 was significantly lower than the normal range (15.51 ng/L, Ref. 119 ± 45 ng/L). LH and FSH was < 0.1 mU/mL and 0.31 mU/ml respectively. The level of TT3 is 1.14 nmol/L (Ref. 1.21–2.66 nmol/L), TT4 is 21.8 nmol/L (Ref. 67–163 nmol/L) and TSH is 1.66 mU/L (Ref. 0.6–4.5 mU/L). The abnormal low level of the above pituitary-related hormone was supportive of the diagnosis of central hypothyroidism and GH deficiency. His level of adrenocorticotropic hormone (ACTH) and cortisol were normal though.

The pituitary MRI showed that he has emptysellasyndrome (2 mm) and low-density shadow in saddle area, indicated of pituitary dysplasia (Fig. 1B1, B2).

## Whole‑exome sequencing (WES) and bioinformatics analysis

For this patient, the main clinical feature was short stature, his intelligence, speech and motor development were normal and his facial features were slightly abnormal. Hormone measurement revealed dysfunction of anterior pituitary. The results of GH response on insulin and arginine hydrochloride testing was supportive of the diagnosis of central hypothyroidism and growth hormone deficiency. So we identified the patient had multiple pituitary hormone deficiencies, therefore we selected WES for genetic detection firstly.

Genomic DNA from the proband and his parents was extracted from peripheral blood leucocytes by using the DNeasy kit (Qiagen). Whole exomes were captured (MyGenostics Inc., Beijing) and sequenced on Illumina NovaSeq 6000 series sequencer (PE150). We applied quality control filters to remove low-quality reads, and performed bioinformatics analysis using an in-house pipeline which included genome alignment with the Burrows-Wheeler Aligner. We annotated database-based MAF and 2015-ACMG Standards and Guidelines based pathogenicity for each generated genetic variant using an online system independently developed by Chigene. Variants with minor allele frequencies of < 0.05 in population databases (such as 1000 Genomes, dbSNP, ESP6500, in-house database (MyGenostics), and EXAC), expected to affect protein coding/splicing or present in the HGMD, would be included into the analysis. As for CNV calls, the total bases for each coding region were calculated using SAMtools, and the average mean depth of the CCDS regions were obtained by using the GATK 'DepofCoverage' command. Then use R to calculate the ratio of each sample compared to the average ratio of the other samples, and use ggplot to visualize the results. Ratios > 1.4 were designated as duplicates and < 0.6 were designated as deletions.

## Genetic results

**Fig. 2:**
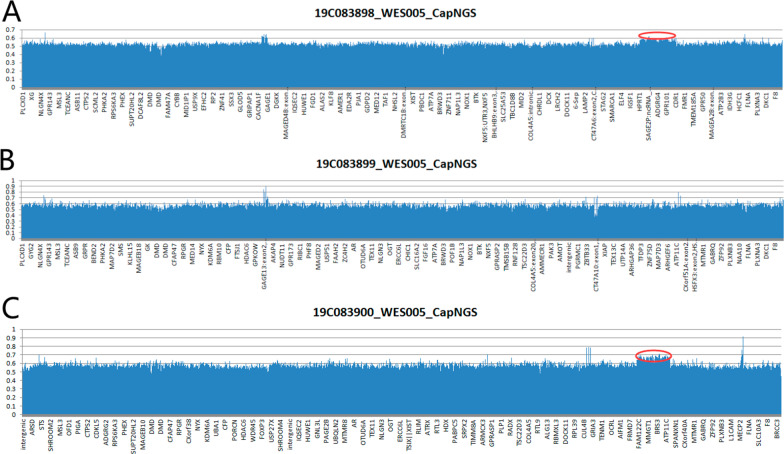
Genetic Analysis diagram of the patient and his parents. The red circle shows 6.180 Mb duplication on Xq26.3q27.1 including *SOX3*, *F9*, and eight other contiguous genes. A represents the patient, B represents his father, and C represents his mother

All the trios-based exomes by the high-throughput sequencing was analyzed (Fig. 2). For genetic screening of all exons, we screened for duplication of X-chromosomes in the patient and his mother. The duplicated segments is 6.180 Mb (Xq26.3 ~ q27.1, 133905314–140085817), involving *ATP11C*, *CD40LG*, *F9*, *DHL1*, *GPR101*, *RBMX*, *SLC9A6*, *SOX3* and *ZIC3*.

## Treatment

**Fig. 3:**
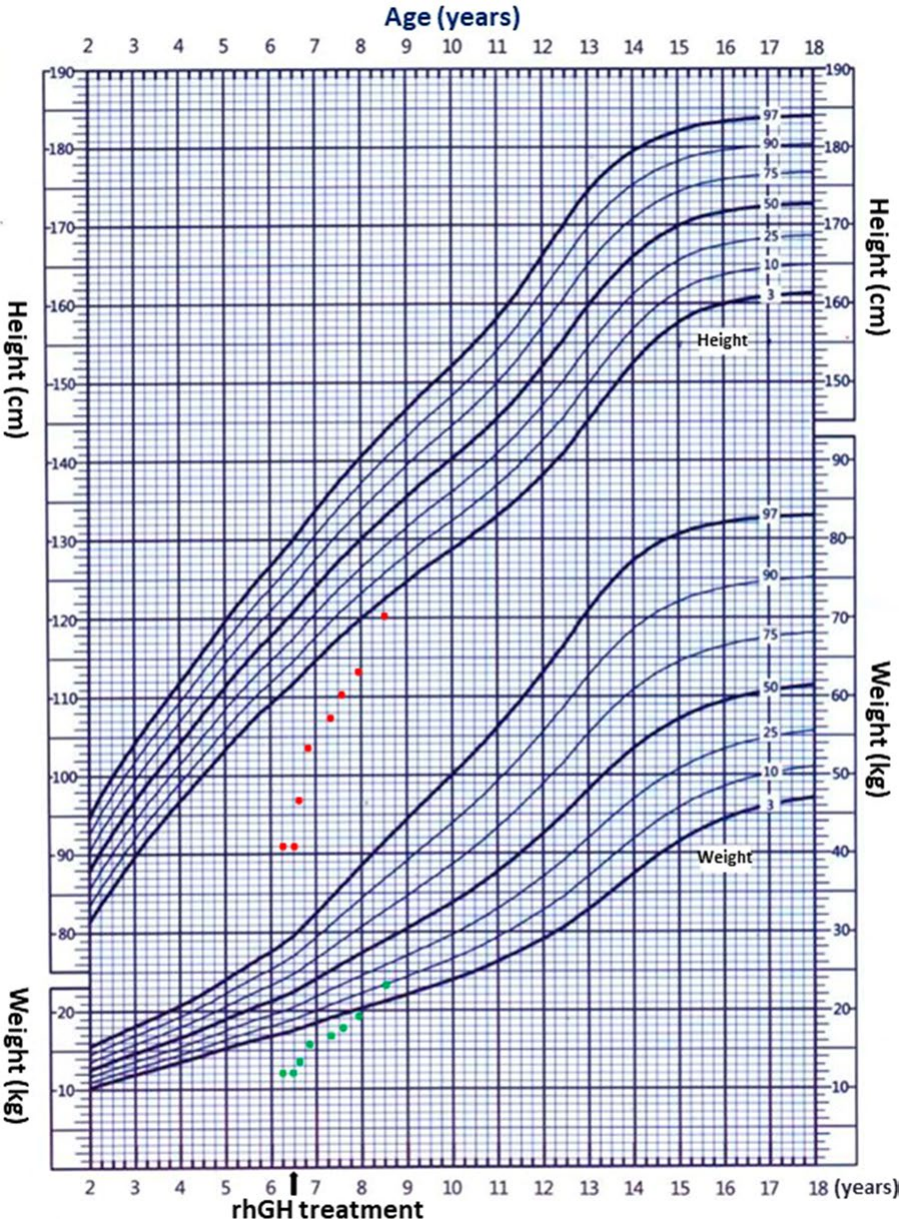
Growth situation of the patient during the 2-year treatment follow-up. The blue line presents a standard growth curve of Chinese boys (2–18 years old). Red and blue dots were the patient’s height and weight during the two-year treatment, respectively

After diagnosis, the patient was first treated with levothyroxine. One month later, the thyroid function was restored to normal, but there was no improvement in height. After adjusting treatment to the recombinant human growth hormone (rhGH, 0.11U/kg/day), the height of the patient was significantly improved (Fig. 3). The growth rate was 2.8 cm/ year before treatment, 17.1 cm/year in the first year of treatment, and 11.9 cm/ year in the second year of treatment. The level of height SDS improved from − 5.87 SDS before treatment to − 3.27 SDS after the first year of treatment and − 1.78 SDS after the second years of treatment (Table 1). The child gained weight slowly during treatment, and 2 years later produced a 10th of the normal child's weight growth curve.

**Table 1:** The growth rate and height in the patient with 2 years treatment

	GV (cm/year)	Height (SDS)
Before treatment	2.8	− 5.78
1-Year treatment	17.1	− 3.27
2-Year treatment	11.9	− 1.78

GV, growth rate

During the 2-year treatment, the dose of rhGH was at 0.114–0.117U/kg/day. The IGF1 level was increased to 113–142 ng/ml. Blood glucose, insulin, ACTH and cortisol was monitored every 3 months, all within the normal range.

## Discussion and conclusions

*SOX3* gene, which belongs to *SOX* family Sry-related High Mobility Group Box, located at Xq27.1. It is a 1,341 nucleotides single exon gene. It is highly expressed throughout development in the pituitary and central nervous system (CNS), and is recognized to have a key role in regulating embryogenesis and CNS development [1]. It is also required for Rathke's capsule morphogenesis and hypothalamic-pituitary axis function [7].

*SOX3* gene variants are associated with hypopituitarism in both animal and patients. Knockout mice experiments indicated that lack of *SOX3* gene can lead to abnormal structure and dysfunction of hypothalamic pituitary axis and special abnormalities in the midline structure of the CNS [7, 8]. Human *SOX3* variant can lead to congenital pituitary dysfunction (clinically manifested as one or several pituitary hormone deficiencies), neural tube defects and other symptoms.

Over-expression or under-expression of *SOX3* can lead to similar clinical phenotypes. Clinical features varied from IGHD or combined pituitary hormone deficiency (CPHD) to panhypopituitarism, as well as anterior hypophysis dysplasia, posterior hypophysis ectopic, abnormal corpus callosum, with or without intellectual impairment [1, 9]. Recently, Arya et al. reported five male patients with Xq27.1 duplication, ranged from 323.8 KB to 11 Mb including *SOX3* gene, diagnosed with congenital dysfunction of hypophysial. Three of them showed multiple pituitary hormone deficiency and two showed IGHD. All five patients presented with small penis or cryptorchidism, abnormalities of pituitary structure or other midline craniocerebral, such as corpus callosum dysplasia, hyaline septum deletion, etc. [10].

Another research reported an 8-year-old boy with 2.1 MB deletion on Xq27.1-q27.2, including the entire *SOX3* gene, had mild mental retardation, language retardation, dysarticulation disorder, behavioral problems, mild facial abnormalities (slightly larger ears, low ear positions, external rotation, protrusion of ear lobes, thin lips, wide nose bridge, low forehead hairline, large hands), excess appetite and overeating. However, the level of GH, ACTH and TSH were at the normal range. Head MRI showed no obvious brain abnormalities at 6 years of age [11]. Dr. Stefano Stagi reported another case [1]. The boy was born as small for gestational age. He presented significantly short stature with lagging bone-age. He has complete growth hormone deficiency, central hypothyroidism, hypogonadism, and decreased ACTH level (but cortisol level was normal). It was different from other case reports that our patient do not have mental retardation or intellectual disability. In our case, the *SOX3* gene duplication originated from his mother, but the mother had no symptoms of this disease, we speculate that it may be related to the random inactivation of the X chromosome. The mechanism of difference in clinical manifestation is unclear yet [9].

Additionally, the prolongation of polyalanine chain is one of the reasons for the decrease of *SOX3* expression. Laumonnier et al. found that 11 alanine repetitions is associated with IGHD, mental retardation, and craniofacial deformity, and Woods et al. found that 7 alanine duplication is mainly manifested as panhypopituitarism and anterior pituitary hypoplasia [5, 6, 12]. Furthermore, incompleteness presence of *SOX3* duplication was reported in literature. Lachlan et al. in 2004 reported a women with functional disomy at Xq27-qter, harboring *SOX3*, but she did not have any signs of GHD [13]. In another study, five women from one family with a 7.5 Mb duplication that including *SOX3*, all exhibited short stature. The author believed that the phenotypic variability may be caused by the altered levels of *SOX3* due to incomplete inactivation of X chromosome variants [14].

Although the *SOX3* gene is structurally similar to the *SRY* gene, it does not have a typical function in sex determination. The HMG DNA-binding domain of *SOX3* is similar to that of the sex-determining gene *SRY* [15], however, the expression of the mouse homologous *SOX3* gene is very low in developing mouse gonads, and *SOX3* variants in mice or humans do not directly result in sexual determination abnormalities [16]. However, overexpression of *SOX3* can up-regulate the expression of *SOX9* through the synergistic effect with *SF1* in the gonadal glands of developing XX mice, thus causing testicular development [17]. Grinspon et al. reported that gain-function variants of *SOX3* lead to *SRY* negative 46, XX DSD [18]. There are two possible mechanisms about it: overexpression of anterior testis genes (e.g., *SOX9*) and underexpression of anterior ovarian/antitestis genes (such as *WNT4* or *RSPOI*). In addition, XX male sex reversal has been reported to be associated with deletions and duplications in the upstream region of *SOX3* [11, 17]. Although loss of function of *SOX3* is not directly involved in sex determination, *SOX3* is a necessary factor to spermatogenesis and gonadal function [19]. In our case, the loss function of *SOX3* gene lead to the left cryptorchidism and right testicular dysplasia.

Over-expression of *SOX3* was also a risk factor for neural tube defects (NTDs), one of the most common congenital defects caused by a combination of genetic and environmental factors. Over expression of *SOX3* may prevent neural tube morphogenesis by undifferentiated neural cell precursors [9]. Duplication of *SOX3* was found in patients with myelomeningococcal, hypophysis and male cognitive impairment [9, 20]. Uguen et al. reviewed 50 patients (44 patients, 6 fetuses) with Xq duplication ranged from 230 kb to 12.5 Mb, all included *SOX3*. Among them, 8 cases (6 fetuses, 2 patients) presented NTDs, indicated that the genetic character of NTDs was incomplete penetrance. In addition, *SOX3* duplication can lead to other abnormalities. Stankiewicz et al. reported a 14-year-old girl with 7.5 Mb duplication on Xq26.2-q27.1 inherited from her mother, presented with dysmorphic features, scoliosis, hearing impairment and amenorrhea. Both the daughter and her mother have severe speech problems with stuttering and dyslalia [14].

So far, there have been no much reports on the treatment of patients with *SOX3* duplication. Arya et al. summarize the general treatment of 4 patients with *SOX3* duplication. However, the dose of the hormone and the change of their growth rate and height has not been reported [10].

In our case, after diagnosis, levothyroxine was given for one month first, and the thyroid function basically returned to normal, but the growth situation did not improve at all. Then rhGH was given, his height, growth rate and height SDS were improved significantly. Gonadal function and long-term prognosis of the patient still need further observation and follow-up. This is the first case of Chinese male patient with multiple hypophysis dysfunction caused by *SOX3* duplication, which will expand the range of phenotypes observed in patients with duplication of *SOX3*.

## Data Availability

The *SOX3* variant can be found in NCBI Nucleotide under the accession number NM_005634. The raw datasets generated and analysed during the current study are not publicly available in order to protect participant confidentiality. The datasets obtained during the current study are available from the corresponding author if the requirements are reasonable.

## Abbreviations

*SOX*: SRY related high mobility group box
IGHD: Isolated GH deficiency
CH: Congenital hypopituitarism
ACTH: Adrenocorticotropic hormone
MRI: Magnetic resonance imaging;
rhGH: Recombinant human growth hormone
CPHD: Combined pituitary hormone deficiency
NTDs: Neural tube defects
